# Targeting Protein Kinase CK2: Evaluating CX-4945 Potential for GL261 Glioblastoma Therapy in Immunocompetent Mice

**DOI:** 10.3390/ph10010024

**Published:** 2017-02-12

**Authors:** Laura Ferrer-Font, Lucia Villamañan, Nuria Arias-Ramos, Jordi Vilardell, Maria Plana, Maria Ruzzene, Lorenzo A. Pinna, Emilio Itarte, Carles Arús, Ana Paula Candiota

**Affiliations:** 1 Departament de Bioquímica i Biologia Molecular, Unitat de Bioquímica de Biociències, Edifici C, Universitat Autònoma de Barcelona, Cerdanyola del Vallès 08193, Spain; Laura.Ferrer@uab.cat (L.F.-F.); Lucia.Villamanan@uab.cat (L.V.); Nuria.Arias@uab.cat (N.A.-R.); Maria.Plana@uab.cat (M.P.); Emili.Itarte@uab.cat (E.I.); Carles.Arus@uab.es (C.A.); 2 Centro de Investigación Biomédica en Red en Bioingeniería, Biomateriales y Nanomedicina (CIBER-BBN), Cerdanyola del Vallès 08193, Spain; 3 Institut de Biotecnologia i de Biomedicina (IBB), Universitat Autònoma de Barcelona, Cerdanyola del Vallès 08193, Spain; 4 Department of Biomedical Sciences, University of Padova, Padova 35131, Italy; jordivilardellvila@gmail.com (J.V.); maria.ruzzene@unipd.it (M.R.); lorenzo.pinna@unipd.it (L.A.P.); 5 Consiglio Nazionale delle Ricerche (CNR), Neuroscience Institute, Padova 35131, Italy

**Keywords:** glioma, preclinical brain tumour, GBM therapeutic target, CK2 inhibitors, CX-4945, metronomic therapy, immune system

## Abstract

Glioblastoma (GBM) causes poor survival in patients even with aggressive treatment. Temozolomide (TMZ) is the standard chemotherapeutic choice for GBM treatment but resistance always ensues. Protein kinase CK2 (CK2) contributes to tumour development and proliferation in cancer, and it is overexpressed in human GBM. Accordingly, targeting CK2 in GBM may benefit patients. Our goal has been to evaluate whether CK2 inhibitors (iCK2s) could increase survival in an immunocompetent preclinical GBM model. Cultured GL261 cells were treated with different iCK2s including CX-4945, and target effects evaluated in vitro. CX-4945 was found to decrease CK2 activity and Akt(S129) phosphorylation in GL261 cells. Longitudinal in vivo studies with CX-4945 alone or in combination with TMZ were performed in tumour-bearing mice. Increase in survival (*p <* 0.05) was found with combined CX-4945 and TMZ metronomic treatment (54.7 ± 11.9 days, *n* = 6) when compared to individual metronomic treatments (CX-4945: 24.5 ± 2.0 and TMZ: 38.7 ± 2.7, *n* = 6) and controls (22.5 ± 1.2, *n* = 6). Despite this, CX-4945 did not improve mice outcome when administered on every/alternate days, either alone or in combination with 3-cycle TMZ. The highest survival rate was obtained with the metronomic combined TMZ+CX-4945 every 6 days, pointing to the participation of the immune system or other ancillary mechanism in therapy response.

## 1. Introduction

Glioblastoma (GBM) is the most common aggressive glial primary brain tumour with an average survival of 14–15 months, even after aggressive treatment [1,2]. Temozolomide (TMZ) plus radiotherapy is the standard therapeutic choice for GBM treatment and, at present, produces the best survival rates [3]. TMZ is an alkylating agent with a mechanism of action based in damaging DNA through methylation of guanine residues, finally leading to cell death. Methylation of the O6 of guanine (O6-MeG) accounts for only about 5%–10% of DNA adducts, but is the primary responsible for the cytotoxic effects of TMZ. The O6-MeG lesion leads to DNA double-strand breaks (DSBs) and subsequent cell death via apoptosis and/or autophagy [4]. In our group, GL261 GBM tumour-bearing mice treated with TMZ showed a survival rate of 33.8 ± 8.7 days [5] versus control mice, 20.0 ± 4.1 days. Unfortunately, cancer stem cells (CSCs) are known to mediate chemoresistance, indicating GBM CSCs persistence even after standard treatment [6]. In addition, cellular exposure to DNA damaging agents (such as TMZ) may cause mutations and clastogenic effects, potentially resulting in additional malignant transformation [7]. Due to the poor outcome and resistance to standard therapy, efficient alternative non-mutagenic treatments are urgently needed for these tumours.

Inhibition of protein kinases has become a standard of modern clinical oncology, and it could improve GBM patients’ survival. Protein kinase CK2 (CK2), an oncogenic protein kinase, contributes to tumour development, proliferation, and apoptosis suppression in cancer [8]. It is a constitutively active serine-threonine kinase and elevated CK2 expression levels have been demonstrated in several cancer types in comparison with normal tissue [9,10,11]. Its overexpression has been also proved in human GBM biopsies compared to adjacent normal tissue [12], regulating signalling pathways involved in tumour cell survival, proliferation, migration and invasion [13]. Furthermore, we have demonstrated that CK2 catalytic subunit (CK2α) expression level was higher in preclinical GL261 GBM tumour and in contralateral brain parenchyma than in wild type C57BL/6 brain parenchyma [14]. These characteristics identify CK2 as an active therapeutic target, and targeting CK2 in GBM treatment could benefit patients. 

Different CK2 inhibitors (iCK2) have been studied for cancer applications, such as 4,5,6,7-tetrabromobenzotriazole (TBB) [15,16] or apigenin (APG) [17,18]. Additionally, a more specific CK2 inhibitor, 5-(3-chlorophenylamino)benzo[c][2,6] naphthyridine-8-carboxylic acid (CX-4945) [19,20,21,22,23] has been reported as the first CK2 inhibitor in clinical stage [24,25]. In vitro studies of breast cancer [21] and studies with an intracranial xenograft murine glioma model [23] have also presented successful results for CX-4945. In addition, it has been reported that CX-4945 decreases GBM initiating cell growth and stemness through β-catenin [26]. Moreover, other promising CK2 inhibitors are in development, such as tetrabromo-deoxyribofuranosyl-benzimidazole (TDB), a dual inhibitor of CK2 and proviral integration of Moloney virus (PIM-1) [27]. These results highlight the relevance of CK2 and its interwoven signalling targets in tumour growth and progression.

Moreover, new therapeutic schedules are being investigated using chemotherapeutic drugs in a metronomic-like approach, referring to administrations of low and equally spaced doses of chemotherapeutics without long rest periods in between [28,29], to try to activate immune responses to potentiate tumour regression and avoid regrowth [29]. For instance, cyclophosphamide (CPA) metronomic therapy has been shown to activate antitumour CD8+ T-cell response, and also induce specific long-term T-cell tumour memory in GL261 GBM tumours growing subcutaneously in immunocompetent mice [30]. 

The use of animal models in tumour research is mandatory in the search for new therapeutic targets due to obvious ethical restrictions related to human patients. One of the most investigated immunocompetent murine brain tumour models is GL261 growing into C57BL/6 mice, used for more than 20 years in different therapy evaluation approaches [5,31,32].

In this work, we have evaluated iCK2 effects over cultured GL261 cells viability to select the best candidates for in vivo preclinical studies. Also, preliminary in vivo work with preclinical GBM was performed, regarding maximum tolerated doses (MTD), tumour targeting effects assessment and survival rate evaluation in longitudinal studies with C57BL/6 mice bearing GL261 tumours treated with iCK2. 

## 2. Results

### 2.1. GL261 Cell Viability under CK2 Inhibition Treatment

**Figure 1:**
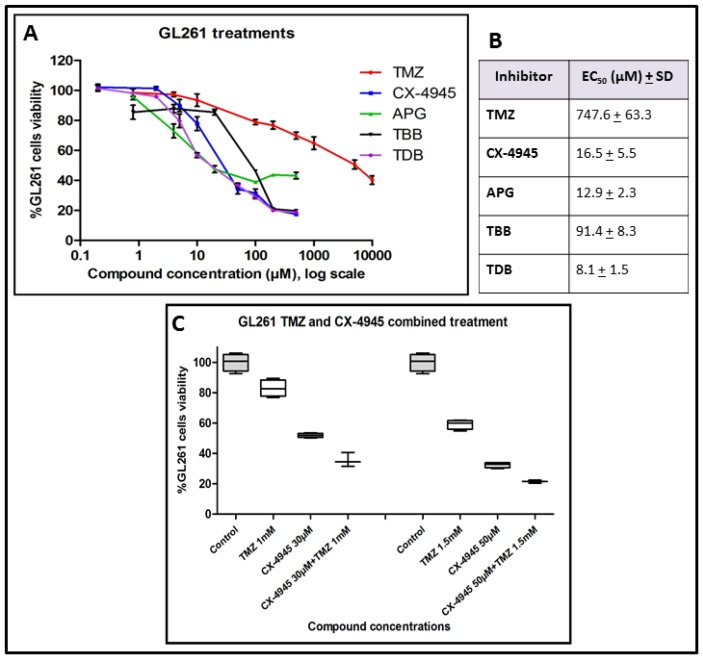
GL261 cells viability after treatment; (**A**) GL261 cells viability (%) “XTT assay” after 72 h of treatment with temozolomide (TMZ): 0 µM, 0.8 µM, 4 µM, 20 µM, 100 µM, 200 µM, 500 µM, 1000 µM, 5000 µM and 10,000 µM, apigenin (APG) and tetrabromobenzotriazole (TBB): 0 µM, 0.8 µM, 4 µM, 20 µM, 100 µM, 200 µM and 500 µM, CX-4945 and tetrabromo-deoxyribofuranosyl-benzimidazole (TDB): 0 µM, 0.2 µM, 2 µM, 5 µM, 20 µM, 50 µM, 100 µM, 200 µM and 500 µM); 100% cell viability was assigned to control cells treated with 0.8% dimethyl sulfoxide (DMSO) (*v*/*v*). Experiments were performed with *n* = 3–9, and mean ± SD values are shown; (**B**) EC_50_ (Half maximal, 50% viability decrease, effective concentration) mean ± SD values obtained with the different treatments to GL261 cells after 72 h of incubation with the drug; (**C**) Boxplot of GL261 cells viability after TMZ and CX-4945 treatment. GL261 cells viability (%) “MTT assay” after 72 h of treatment. On the left side, control (*n* = 4), CX-4945 30 µM (*n* = 4), TMZ 1 mM (*n* = 4) and CX-4945 30 µM plus TMZ 1 mM (*n* = 3); on the right side, control (*n* = 4), CX-4945 50 µM (*n* = 4), TMZ 1.5 mM (*n* = 4) and CX-4945 50 µM and TMZ 1.5 mM (*n* = 3); 100% cell viability was assigned to control cells treated with 0.8% DMSO (*v*/*v*). As both experiments were performed at the same time, controls were acquired only once and, accordingly, the same control cells results are shown for both experimental conditions. Experiments were performed with *n* = 3–4, and mean ± SD values are shown. Boxplot (the limits of the box represent quartiles 1 (Q1) and 3 (Q3) of the distribution, the central line corresponds to the median (quartile 2). The whiskers symbolize the maximum and minimum values in each distribution.

Cultured GL261 cells sensitivity to different treatments was assessed. Figure 1A,B showed a low half maximal effective concentration (EC_50_) (12.9 ± 2.3 µM) for APG, similar to CX-4945 (16.5 ± 5.5 µM), but the final viability reached with APG was only up to 40% of the initial value. Instead, TBB and CX-4945 both decreased viability to about 20% of the initial value. Regarding to TBB, a concentration of 91.4 ± 8.3 µM produced a 50% viability decrease, whereas for CX-4945, a concentration of 16.5 ± 5.5 µM produced the same results. In the case of TDB, it showed the lowest EC_50_ (8.1 ± 1.5 µM), and the final viability obtained was similar to CX-4945. TMZ EC_50_ was 747.6 ± 63.3 µM. 

Additionally, an in vitro experiment with combined treatment of GL261 cells was outlined, and as it can be seen in Figure 1C, we could demonstrate that the combined administration of CX-4945 and TMZ to GL261 cultured cells presented an increased efficacy in comparison with treatments of single substances alone. TMZ alone reduced cell viability to 82.8% ± 5.6% (at 1 mM) and 59.2% ± 3.2% (at 1.5 mM) in comparison to controls, whereas CX-4945 alone reduced cell viability to 52.0% ± 1.4% (at 30 μM) and 31.9% ± 2.1% (at 50 μM). The combined administration of both therapeutic agents resulted in a cell viability reduction to 35.6% ± 4.7% (TMZ 1 mM + CX-4945 30 μM), and to 21.5% ± 1.0% (TMZ 1.5 mM + CX-4945 50 μM) in comparison to controls, being clearly superior to the efficacy of each substance separately. Concentrations chosen were above the EC50 (Figure 1A,B), to ensure enough cell viability reduction. In the remainder of this study, we decided to focus first on CX-4945, one of the two most effective CK2 inhibitors tested on GL261 cell viability (Figure 1) because unlike the other one, TDB, it has been already used in clinical trials [24,25].

### 2.2. CK2 Activity in GL261 Cells Treated with CX-4945

**Figure 2:**
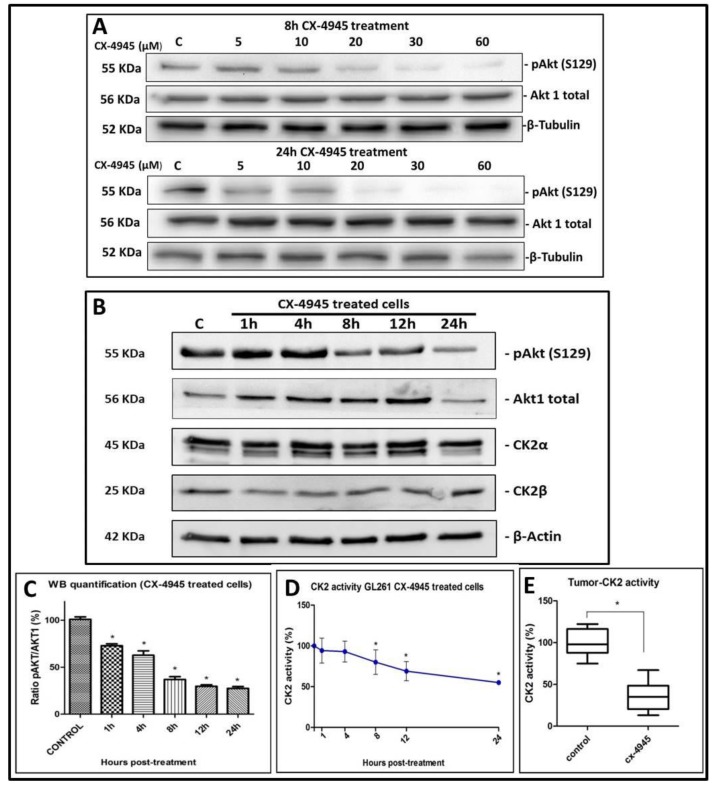
CK2 activity in GL261 cells and tumour samples. (**A**) Western blot for GL261 cell protein extracts (25 µg) treated with increasing doses of CX-4945 (from left to right: control (C) and CX-4945 treated cells 5 µM, 10 µM, 20 µM, 30 µM and 60 µM). This experiment was performed with *n* = 1 for each condition and for 8 h (upper part) or 24 h (lower part). p-Akt(S129), Akt1 total and β-Tubulin proteins were analysed; (**B**) Western blot for GL261 cell protein extracts (25 µg) treated with 67.2 µM CX-4945 (from left to right: control (C) and CX-4945 treated cells for 1 h, 4 h, 8 h, 12 h and 24 h). The experiment was performed with *n* = 3 for each condition. p-Akt(S129), Akt1 total, CK2α, CK2β and β-Actin proteins were analysed; (**C**) Quantification of western blot (WB) for GL261 cell protein extracts (25 µg) treated with CX-4945 (*n* = 3 per each condition). Ratio (%) of p-Akt(S129) content divided by Akt1 total content, while the control values (C) for this ratio are taken as the 100% start value. * = *p* < 0.05 for Student’s *t*-test for the comparison of control and treated groups; (**D**) CK2 activity measured on a CK2-specific synthetic peptide in lysates from GL261 cells treated with 67.2 µM CX-4945 (*n* = 3 for each condition). Treatment during 1 h, 4 h, 8 h, 12 h and 24 h. * = *p* < 0.05 for Student’s *t*-test for the comparison of control (100% initial value) and treated groups; (**E**) Boxplot of CK2 activity in CX-4945 treated mice compared to control mice. CX-4945 was administered to treated mice during 3 days (a total of 150 mg/Kg/day split into two administrations per day) and mice were euthanized 2 h, 6 h and 24 h after the last CX-4945 administration. As no CK2 activity differences (*p* > 0.05) were detected between euthanization time points (2 h, 6 h and 24 h), they were grouped in a single CX-4945 treated group. (* = *p* > 0.05 for Student’s *t*-test for the comparison of control and treated groups). CK2 activity was measured on tissue homogenates by means of radioactive assays towards a CK2-specific peptide. Boxplot features as in Figure 1 legend.

CK2 activity was analysed in GL261 cells treated with CX-4945 and in control, non-treated cells. As a reporter of endocellular CK2 activity, the phosphorylation state of the well-known CK2 target Akt (S129) [33] was analysed. In Figure 2A, p-Akt (S129) normalized to total Akt1 expression was obtained (at 8 h and 24 h post-treatment) and CX-4945 presented a dose-scale response, being p-Akt (S129) lower when higher concentrations of the inhibitor were applied. In addition, Figure 2B,C shows that p-Akt (S129), normalized to total Akt1, is significantly (*p <* 0.05) less phosphorylated in GL261 cells treated with 67.2 µM CX-4945 compared to control cells. No differences were found for CK2α and CK2β expression (*p* > 0.05) between treated and non-treated cells. CK2 activity was also measured in cell lysates, exploiting a highly specific peptide substrate [34] and significant differences (*p <* 0.05) were found between CX-4945 treated cells (Figure 2D) and control cells (pre-treatment). These results indicate that CX-4945 reduces endogenous CK2 activity when used to treat cultured GL261 cells, but not the total amount of CK2 subunits present in those cells. 

### 2.3. CX-4945 Mice Tolerability 

Before starting longitudinal in vivo treatment experiments, tolerability evaluation was performed for CX-4945 and TMZ. As it can be observed in Figure S1A, in the first phase, an MTD of 920 mg/kg was estimated for TMZ and of 1200 mg/kg for CX-4945. These MTD values were chosen because the doses of 1840 mg/kg (TMZ) and 2400 mg/kg (CX-4945) produced toxicity/adverse effects to the treated mice. In phase 2, when *n* = 3 mice where administered with a dosage of 920 mg/kg of TMZ, 9 days after this single TMZ administration, noticeable body weight decrease was detected in all mice. For this reason, the experiment was repeated with an *n* = 3 at the next lower dose, 480 mg/kg (Figure S1B). A similar situation was observed for CX-4945 when 1200 mg/kg of CX-4945 were administered per *n* = 3, one mouse was found dead, the day after administration so the experiment was repeated at 600 mg/kg (Figure S1C). These results indicate that the MTD (acute dose) is 480 mg/kg for TMZ and 600 mg/Kg for CX-4945, under our experimental conditions. 

### 2.4. CK2 Activity in CX-4945 Treated Mice

In preliminary in vivo target validation studies, CK2 activity was found significantly (*p <* 0.05) reduced in all samples analysed, in comparison to controls (Figure 2E). Values obtained at the different time points (2 h, 6 h and 24 h) did not present significant differences when compared (*p* > 0.05), and results were grouped in *n* = 6 treated and *n* = 6 control mice. These results indicate that CX-4945 successfully reached tumours and exerted the expected effect on its target.

### 2.5. Metronomic Longitudinal Treatments with CX-4945 and/or TMZ in Tumour-Bearing Mice

**Figure 3:**
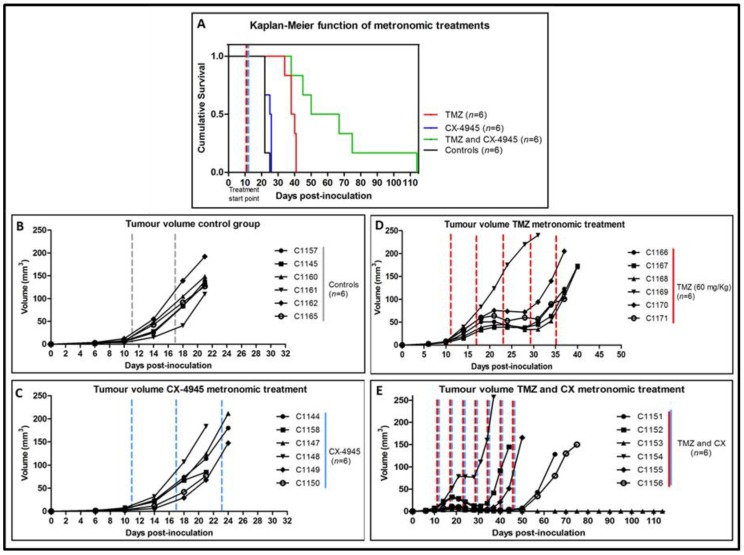
Tumour volumes and survival rates for 6-day metronomic treatment. (**A**) Survival Kaplan-Meier curve for metronomic CX-4945 treated mice (*n* = 6), blue line, metronomic TMZ treated mice (*n* = 6), red line, CX-4945 and TMZ metronomic treated mice (*n* = 6), green line, and control mice (*n* = 6), black line. Significant differences (*p* < 0.05) were observed between all treatment groups analysed in comparison with control mice; (**B**) Tumour volumes of control mice (*n* = 6); (**C**) Tumour volumes of metronomic CX-treated mice (*n* = 6); (**D**) Tumour volumes of metronomic TMZ-treated mice (*n* = 6); (**E**) Tumour volumes of metronomic CX and TMZ- treated mice (*n* = 6). Significant differences were found between groups (*p* < 0.05) when comparing control mice with treated mice, and when comparing different treatments between them (TMZ, CX-4945 and combined TMZ and CX-4945). The dashed lines indicate therapy administration points. Cxxxx corresponds to a unique alpha-numeric animal identifier code in the GABRMN group.

Three metronomic (every 6 days) administration treatments were performed: CX-4945 (*n* = 6), TMZ (*n* = 6) and a combination of CX-4945 and TMZ (*n* = 6), Figure 3. For the CX-4945 metronomic treatment, a survival rate of 24.5 ± 2.0 days was found, whereas for TMZ treatment, the survival rate was 38.7 ± 2.7. For the combined TMZ and CX-metronomic treatment, a provisional survival rate (right censored data) of 54.7 ± 11.9 days was found (three mice were still alive at day 65 p.i.). All groups offered significantly higher survival rate (*p <* 0.05) compared to control mice group (22.5 ± 1.2 days), Figure 3A. Besides, TMZ alone produced better survival than CX-4945 alone, while combined TMZ and CX-4945 was better than any of the two alone. Moreover, tumour volume evolution (Figure 3B–E) was significantly different (*p <* 0.05) when comparing treated groups with control group. Body weight was also inspected every day (Figure S3D). At the time of comparing the different treated groups, significant differences were found regarding weight, tumour volume evolution and survival rate, being the best results always obtained with the metronomic treatment combining TMZ with CX-4945.

### 2.6. Non-Metronomic Longitudinal Treatments with CX-4945 and/or TMZ in Tumour-Bearing Mice

Despite having demonstrated that CX-4945 produces a better effect in GL261 implanted mice when administered in combination with TMZ and administered every 6 days, additional results of longitudinal mice experiments must be taken into account to better understand its causes, as in some cases, antagonistic effects can be observed.

**Figure 4:**
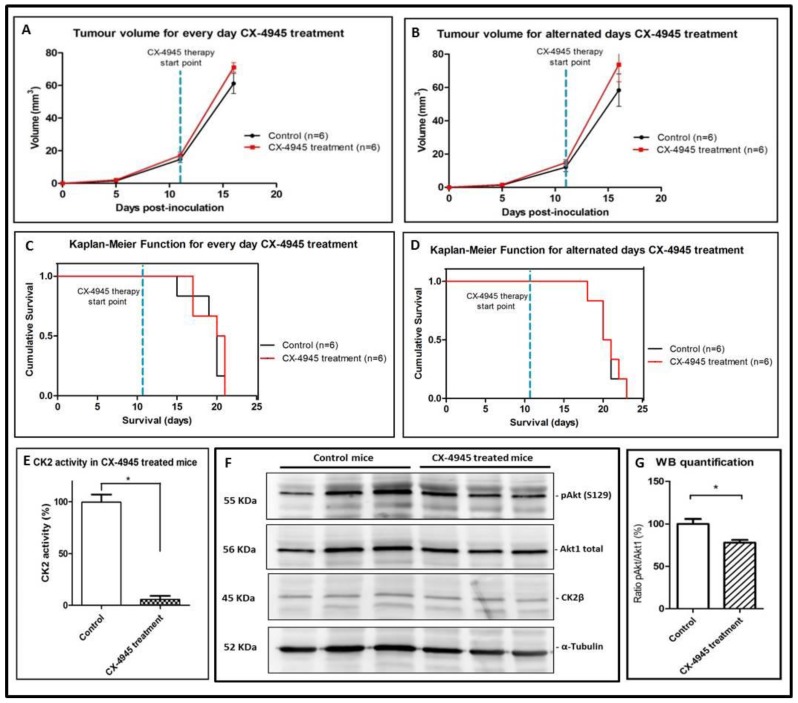
Tumour volumes, survival rates and CK2 activity and expression in CX-4945 treated mice. Tumour volumes (recorded at days 5, 11 and 16 p.i.) of treated (*n* = 6, black line) and control non-treated bearing tumour mice (*n* = 6, red line) for (**A**) CX-4945 treated every day GL261 implanted mice and (**B**) CX-4945 treated alternated days GL261 implanted mice. No significant differences were observed between groups (*p* > 0.05). The dashed blue line indicates the CX-4945 therapy start point; (**C**) Survival Kaplan-Meier curve for CX-4945 treated every day mice (*n* = 6) and control mice (*n* = 6); (**D**) Survival Kaplan-Meier curve for CX-4945 treated alternated days mice (*n* = 6) and control mice (*n* = 6). No significant differences were found between groups (*p* > 0.05). The dashed blue line indicates the CX-4945 therapy start point; (**E**) Tumour CK2 activity (%) in mice treated with CX-4945, *n* = 3, compared with control mice, *n* = 3. (* = *p* < 0.05 for Student’s *t*-test for the comparison of control and treated groups); (**F**) Western blot for tumour total protein homogenate (40 µg) from different mice treated with CX-4945, *n* = 3, compared with control mice, *n* = 3. p-Akt(S129), Akt1 total, CK2α, and α-tubulin proteins were analyzed; (**G**) Quantification of Western blot for tumour total protein homogenate (40 µg) from mice treated with CX-4945, *n* = 3, compared with control mice, *n* = 3. Ratio (%) of p-Akt (S129) content divided by Akt1 total content. * = *p* < 0.05 for Student’s *t*-test for the comparison of control and treated groups.

In this respect, CX-4945 treatment (either every day or alternated days administration at the classical dose/schedule described by others [21,22,23]) was performed in GL261 tumour-bearing mice, and no improvement was detected regarding tumour evolution (Figure 4A,B) or survival rate (*p* > 0.05, Figure 4C,D) in comparison with control mice. Figure 4C,D show the survival rate for every day CX-4945 treatment (20.5 ± 2.0 vs. 20.0 ± 2.1 days for control mice) and for alternated days CX-4945 treatment (20.5 ± 1.8 days vs. 20.5 ± 1.6 days for control mice). Body weight was inspected every day and no significant differences (*p* > 0.05) were observed between groups (Figure S3A). Examples of T_2w_ images for CX-4945 treated mice are shown in Figure S4. To ensure that CX-4945 reached the tumour, even when no effect on survival could be detected, 6 arbitrarily chosen tumour samples from the CX-4945 treatment every day (*n* = 3 treated, *n* = 3 controls) were analysed for CK2 activity and p-Akt (S129) WB. The CK2 activity was more than seventeen-fold reduced in CX-4945 treated tumour compared to control tumour after 10.0 ± 2.0 days of treatment (Figure 4E) and p-Akt (S129)/Akt1 ratio (Figure 4F,G) was found around 20% reduced in CX-4945 treated mice, indicating that CX-4945 had reached the desired target and inhibited CK2 activity, despite no increase of the survival rate was observed.

**Figure 5:**
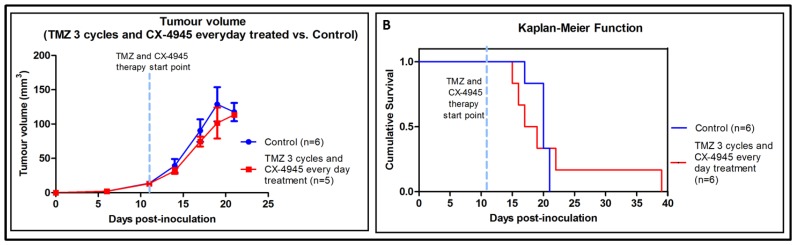
Tumour volume and Kaplan-Meier survival curve of mice treated with TMZ combined with CX-4945; (**A**) Tumour volumes (recorded at days 6, 11, 14, 17, 19 and 21 p.i.) of control GL261 implanted mice (*n* = 6, blue line) and combined TMZ-CX-4945 cycles in GL261 implanted mice (*n* = 5, red line). The dashed blue line indicates TMZ and CX-4945 therapy start point. No significant differences were observed between groups for comparisons of control and treated mice tumour volumes at each time point (*p* > 0.05). C984 has been excluded from tumour volume comparisons because it has been proven an outlier with Grubb’s and Dixon’s tests; (**B**) Survival Kaplan-Meier curves for TMZ and CX-4945 treated mice (*n* = 6) and control mice (*n* = 6). No significant differences were found between groups (*p* > 0.05). The dashed blue line indicates TMZ and CX-4945 therapy start point.

An additional experiment with three cycles TMZ and CX-4945 combined administered every day, produced significantly worse results than TMZ treatment alone (see [5] and Figure S5). In other words, CX-4945 treatment in vivo, in these conditions, seems to inhibit the beneficial effect produced by TMZ. Tumour volume curves are shown in Figure 5A. As stated in Figure S3B, three out of the six treated mice died around day 16 p.i., without noticeable weight reduction. Control mice weight evolution can be observed in Figure S3C. To compare survival rates between groups, Kaplan-Meier survival curves were elaborated, and no significant differences were found between TMZ 3 cycles + CX-4945 every day treated group and control group (*p* > 0.05, Figure 5B). The average survival rate was 21.3 ± 9.0 days for treated mice (TMZ 3 cycles + CX-4945 every day) vs. 19.8 ± 1.5 days for control mice, while for TMZ three cycles only treated mice from previous work (Figure S5) average survival time found was 33.9 ± 11.7 days. In addition, no significant differences were found when comparing TMZ 3 cycles+CX-4945 every day and CX-4945 everyday alone (survival rates of 19.8 ± 1.5 vs. 20.5 ± 2.0 days, respectively). C984 mouse was proven to be an outlier both in Grubbs’ and Dixon’s tests for single outliers (survival rate of 39 days, *p <* 0.05), but still it was maintained for survival analysis calculations. Overall average survival for control (untreated) GL261 harbouring mice was 20.8 ± 1.8 days, *n* = 18, while previous work from our group had obtained 21.5 ± 3.7 days, *n* = 61 (Figure S5), without significant difference (*p* > 0.05) with the present cohort of mice.

## 3. Discussion

### 3.1. Effect of iCK2 on GL261 Cultured Cells

GL261 cultured cells sensitivity was variable for the different iCK2 evaluated. CX-4945 decreased cell viability to about 20% of the control value. The EC_50_ found for CX-4945 (16.5 ± 5.5 µM) is similar to the EC_50_ found in breast cancer cell lines by others [21], who reported values between 1.7 and 20 μM. The lowest EC_50_ (8.1 ± 1.5) was found for TDB, concurring with a previous study [27], where a half maximal cell death concentration (DC_50_) of 2.45 ± 0.84 µM was observed for the human HeLa cell line and of 3.45 ± 0.2 µM for human T lymphoblastoid cells. The standard GBM chemotherapeutic agent, TMZ, reached the EC_50_ at 747.6 ± 63.3 µM, which agrees with high values of half maximal inhibitory concentration (IC_50_) found by others [35].

In addition, the combined TMZ and CX-4945 treatment carried out with GL261 cells in vitro presented increased efficacy regarding cell viability reduction, in comparison with both therapeutic agents administered separately (Figure 1C). The low EC_50_ of CX-4945 and TDB, combined with a significant decrease in cell viability, indicated that they should be suitable for future preclinical evaluation with GL261 tumours in vivo. CX-4945 was eventually chosen because it has been already described in clinical and preclinical studies [24,25].

As expected, we observed a significant reduction of CK2 activity in CX-4945 treated GL261 cells (Figure 2D). This was accompanied by the decrease of the phosphorylation of the CK2 target Akt (S129) (Figure 2B,C), similarly to [22] in prostate PC3 cancer cells. In addition, the Akt phosphorylation was found reduced as the CX-4945 concentration was increased, in a dose-dependent manner (Figure 2A). It has been described that the antiapoptotic effect of CK2 can be partially mediated by upregulation of the Akt/PKB pathway [33], thus the reduced viability found in GL261 cultured cells treated with CX-4945 can be also related to the down-regulation of this pathway, although the involvement of other pathways could also have an effect in the overall results observed [36]. These results reinforced the idea that CX-4945 could be a promising candidate for non-mutagenic brain tumour therapy in our preclinical GBM model. 

### 3.2. Effects of CX-4945 in In Vivo Studies

In in vivo studies, CX-4945 was described to inhibit the activation of STAT-3, NF-κB p65 and Akt, in nude mice with intracranial human GBM X1046 xenografts [23]. In our work, we could demonstrate that 3 days of CX-4945 administration to GL261 tumour-bearing mice caused a decrease in CK2 activity of tumour samples analysed to 35% of control values (Figure 2E). This confirms that CX-4945 reached the target organ and caused the expected effect, as also reported in [23], although no further detailed study of the affected pathway was performed in our case. Regarding CX-4945 pharmacokinetics, described extensively elsewhere [37], it has a long half-life (more than 5 h) and high oral bioavailability (20%) in mice, without detectable mutagenicity, genotoxicity or cardiac toxicity. The results described in our work agree with this, showing that CX-4945 was well tolerated in mice as assessed by minimal changes in body weight during the course of treatment compared to control vehicle (Figure S3A).

### 3.3. Non-Metronomic CX-4945 Longitudinal In Vivo Studies

Although in vitro results indicated that CX-4945 should be a promising candidate for preclinical GBM therapy, unexpectedly, the non-metronomic in vivo treatment of GL261 tumour-bearing mice did not produce a better outcome. No tumour growth arrest and no survival improvement were detected (Figure 4A–D). This is in contrast to a previous in vivo study [23] with intracranial human xenografts treated with CX-4945 which showed significant effects in mice survival (50.2–67.8 days for treated mice, versus 35.6–40.4 days for control mice). However, there are relevant differences between work reported in [23] and our work, which could explain, at least partially, the differences observed. In [23], athymic nude mice were used, while in our experiment, immunocompetent C57BL/6 mice have been used, because we consider that they mimic better the clinical patient situation. Moreover, despite authors in [23] inoculated more cells for tumour generation (5 × 10^5^ cells vs. 10^5^ cells, in our study), their tumour growth pattern was much slower (survival rate for their control mice, 35.6–40.4 days, whereas our control mice survived 17–21 days). In this sense, it is widely accepted that small, slow-growing tumours are easier to treat than established, fast-growing tumour [38,39].

It is also worth mentioning that longitudinal in vivo studies reported by other authors did not present satisfactory results with CX-4945 alone in the same doses used in this work, although they studied other tumour types [36]. In this sense, using a drug combination for therapy involving CX-4945 was already suggested by authors in [36,40], with an additive effect recorded, which is also in line with our decision to study a drug combination (CX-4945 and TMZ) in order to perhaps improve the therapy response results produced by CX-4945 alone. 

### 3.4. Non-Metronomic Combined CX-4945 and TMZ Longitudinal In Vivo Studies

Thus, the unexpected result obtained with the in vivo longitudinal study using CX-4945 alone in GL261 tumour-bearing mice lead us to hypothesize that a combined therapy with CX-4945 superimposed with TMZ could produce better results. The TMZ treatment in preclinical GBM mice has been proved useful in our group [5] with an average survival of 33.9 ± 11.7 days for treated animals (see also Figure S5), significantly higher than control mice (21.5 ± 3.7 days). TMZ cytotoxicity is predominantly mediated by O6-methylguanine (O6-MG) DNA lesions, which are repaired by the DNA repair protein O(6)-methylguanine-DNA methyltransferase (MGMT) [41]. Consequently, GBM patients whose tumours express low MGMT level, due to promoter hypermethylation, are more responsive to TMZ based therapy [42,43]. Protein Kinase CK2 is a novel interaction partner of JAK1/2, potentiating janus kinase (JAK) and signal transducer and activator of transcription (STAT-3) activation [44]. A CK2 inhibitor could reduce STAT-3, which has been implicated in the resistance of GBM to TMZ, downregulating MGMT and diminishing TMZ resistance [45], highlighting the potential of use of iCK2 combined with standards of care like TMZ. Nevertheless, the combined treatment (standard 3-cycle TMZ described by our group [5] superimposed with non-metronomic schedule for CX-4945) did not produce the expected improvement, except by case C984, classified as an outlier (Figure S3C), which followed an evolution pattern similar to TMZ-treated cases described in [5]. Except for this case, the combined TMZ+CX-4945 therapy showed similar results than control mice (survival of 17.8 ± 2.8 days for treated mice vs. 19.8 ± 1.5 days for control mice), reversing the beneficial effect of TMZ 3 cycles treatment. However, the combined TMZ and CX-4945 treatment carried out with GL261 cells in vitro presented increased efficacy regarding cell viability reduction, in comparison with both therapeutic agents administered separately (Figure 1C). This reinforced the idea that an increased efficacy, instead of an antagonistic effect, should have been observed in vivo with the combined therapy. 

One of the possible explanations for those results could be due to dramatic CX-4945 ‘off target’ effects, e.g., leading to strong splicing inhibition [46]. We cannot discard that these effects, rather than CK2 inhibition, are responsible for reversing TMZ efficacy. Another explanation could be related to the role of the immune system in therapy response [47] (see below), which would help to explain why a synergistic effect was observed with GL261 treated in vivo cells but it was not seen in our early non-metronomic in vivo approach. 

### 3.5. Metronomic CX-4945 and/or TMZ Longitudinal In Vivo Studies and Possible Implication of the Immune System

The disappointing results obtained with non-metronomic administration schedules of CX-4945 both alone or in combination with TMZ lead us to raise the hypothesis that a possible interference with the host immune system was taking place, and it was the cause of the unfavourable outcome. Thus, we decided to move to a metronomic approach to discard or confirm this hypothesis. The so-called “metronomic therapy” [28], referring to equally spaced, low doses of chemotherapeutic drugs without extended rest periods, has been studied by several groups in the preceding years. Also, new therapeutic regimens with conventional drugs have been evaluated in order to activate immune responses that enhance tumour regression and prevent tumour regrowth. Recent studies with cyclophosphamide (CPA) metronomic therapy proved that this type of administration not only activates antitumour CD8+ T-cell response, but also induces long-term, specific T-cell tumour memory in GL261 tumours growing subcutaneously in immunocompetent mice [30,48]. These authors have also proven that a 6-day intermittent was the optimum timing for this therapy and this could agree with the 7-day cycle for immune cell recruitment described in [49], and accordingly this was the schedule chosen for our metronomic treatments. Indeed, the metronomic (every 6 days) therapy carried out in our study offered a better mice outcome, being the best results produced by CX-4945 and TMZ combined (54.7 ± 11.9 days) metronomic therapy, which was better than CX-4945 or TMZ metronomic therapies alone. These results reinforce the idea of the role of the immune system in therapy response [47] in GBM, and could explain the variation in our results depending on the therapy administration protocol used. Regarding the immune system cycle involved in tumour response, we should consider that the cytotoxic T-lymphocytes (CTLs) have a relevant role in the defence against cancer recognizing antigens presented on the surface of transformed cells, following a complex cycle described in [50]. Also, the whole cycle for immune cell response could take around 7 days in mouse brain [49], which is in agreement with the 6-day interleave that we have used in our work. It was also described the need of a functional CD5-dependent CK2 signalling for efficient differentiation of naive CD4+ T cells into Th2 and Th17 cells [50,51], involved in monocytes differentiation into dendritic cell subsets [52]. Moreover, authors in [53] have recently described impairment of Th17 cells development by CK2 inhibition with CX-4945 in a C57BL/6 mouse model of experimental autoimmune encephalomyelitis. Accordingly, CK2 inhibition (which could take place in every day or alternated days CX-4945 administration) could impair proper attraction of immune response triggered by immunogenic cell death signals. In this respect, several authors have described immunogenic death caused by TMZ therapy in GBM [54,55] and it could explain the reversal of beneficial effects observed in the non-metronomic combined therapy. Additionally, CK2 inhibition in vitro has been shown to compromise normal T-cell viability of cultured peripheral blood T-lymphocytes harvested from chronic lymphocytic leukemia patients [56], while other authors [36] demonstrated cytotoxic effects of CX-4945 administration, alone, in head and neck cancer cultured cell lines and xenograft models. Further work will be needed to clarify, in vitro and in vivo, the extent of immunogenic cell death produced by TMZ and CX-4945, alone or in combination, in the GL261 GBM model.

In summary, it is tempting to speculate that when CX-4945 is administered every day or in alternated days, it could cause an impairment of immune system elements needed for tumour response. Furthermore, when it is administered combined with TMZ (three cycles of therapy), CX-4945 reversed the beneficial effect of TMZ. When TMZ and CX-4945 are administered in a metronomic scheme every 6 days, the activation of the immune cell recruitment and response, which can take around 7 days [49], would not be significantly compromised. Accordingly, a word of caution should be said when treating immunocompetent preclinical tumours with CX-4945: the continued administration of a drug could impair the proper attraction of immune response contributing to therapeutic effects being evaluated. 

## 4. Materials and Methods

### 4.1. GL261 Cells 

GL261 mouse glioma cells were obtained from the Tumour Bank Repository at the National Cancer Institute (Frederick, MD, USA) and were grown as previously described in [57]. 

### 4.2. Cell Viability Assay

GL261 cells were plated in 96-well multiwell plates (Sigma Aldrich, Madrid, Spain) and allowed to adhere for 24 h before adding drugs to the medium (TMZ, APG, TBB, CX-4945 and TDB). Controls included cell culture medium and 0.4%–0.8% range of dimethyl sulfoxide (DMSO). After 72 h of drug exposure, cell viability was measured using XTT or MTT Assay (Sigma Aldrich). Half maximal effective concentration (EC_50_) was calculated using GraphPad Prism software [58] See Supplementary Material file for further details. 

### 4.3. Antibodies

CK2α-subunit (*dil. 1:1000*) rabbit antiserum was raised against (376–391) region of human protein, corresponding to the specific C-terminal sequence. CK2β (*Monoclonal, dil. 1:750, rabbit, Ref Ab76025*) and Phospho-Akt (S129) (*dil. 1:1000, rabbit, Ref 133458*) were purchased from Abcam (Cambridge, UK). β-actin (*dil.1:2000, mouse, Ref A2228*) and α-tubulin (*dil.1:2000, mouse, Ref A5441*) were obtained from Sigma-Aldrich, and β-tubulin (*dil. 1:1000, rabbit, Ref 2146*) and Akt1 (*dil 1:500, rabbit, Ref C3H10*) were purchased from Cell Signalling Technology (Beverly, MA, USA). Secondary antibodies towards rabbit and mouse IgG (*dil.1:2000*), conjugated to horse radish peroxidase, were purchased from PerkinElmer (Waltham, MA, USA).

### 4.4. Target Evaluation in GL261 Cultured Cells with CX-4945 Treatment

For the CK2 expression and activity studies, a total of 18 flasks (75 cm^2^) of GL261 cells were cultured (*n* = 3 as controls, *n* = 3 as CX-4945 treatment during 1 h, 4 h, 8 h, 12 h and 24 h). Cells were cultured in flasks until 50% confluence and at that moment, CX-4945 was added to the “treatment” flasks, and an equal amount of the vehicle (DMSO) was added to the control cells. The CX-4945 concentration used was 67.2 µM (four times the EC_50_, as stated by other authors [59]). Still, this concentration was included in the range of maximum CX-4945 effect as it can be seen in Figure 1. An in vitro dose-response study was also conducted in cultured GL261 cells in order to check for increasing target effects. For this, 300,000 cells/well were plated in 6-well plates and concentrations of CX-4945 of 0, 5, 10, 20, 30 and 60 µM were added. Treatments were performed during 8 and 24 h. Therapeutic agent preparations (CK2 inhibitors and TMZ) can be found in the Supplementary Materials file.

### 4.5. Animal Model for In Vivo Studies

A total of 60 C57BL/6 female wild type mice weighting 21.2 ± 1.3 g were used for this study. They were obtained from Charles River Laboratories (Charles River Laboratories International, L’Abresle, France) and housed in the animal facility of the Universitat Autònoma de Barcelona. All studies described in this paper were approved by the local ethics committee (Comissió d’Ètica en Experimentació Animal i Humana (CEEAH), [60]), according to the regional and state legislation (protocol DMAH-8236/CEEAH-2785). Tumours were induced in C57BL/6 mice by intracranial stereotactic injection of 10^5^ GL261 cells with 4 μL of RPMI (Roswell Park Memorial Institute) medium in the caudate nucleus, as previously described by us [61]. Mice were weighted every day or at alternated days (for metronomic treatments) and tumour volumes were followed using T_2_-weighted MRI acquisition as in [5] the day 6 and 10-11 after implantation. Mice with most homogeneous weights and tumour sizes were chosen to make experimental groups after randomization (usually *n* = 6 per studied condition), and therapy started. The volume and weight averages did not show significant differences (*p* > 0.05) between the experimental groups (Tables S1–S4).

### 4.6. CX-4945 and TMZ Tolerability Assay

Two phases were performed for this study, based in the work described in [62]: in phase 1, *n* = 1 was used and the starting TMZ dose was the one described by us (60 mg/kg/day) [5], and for CX-4945, the dose was the one described in [22,59] (150 mg/kg/day), as no adverse effects have been observed with these doses. Administrations were performed with an oral gavage. Doses were increased (Table S5) until detection of toxicity symptoms (Table S6). Once MTD was estimated in this first phase, a second phase took place and a group of *n* = 3 mice were administered with the calculated MTD. Mice follow-up (weight + welfare parameters for up to 30 days) was carried out every day (Figure S1). 

### 4.7. Use of iCK2 in Tumour-Bearing Animals

#### 4.7.1. Preliminary Studies of Target Validation

CX-4945 therapy was administered to tumour-bearing mice (*n* = 2) during three days (150 mg/kg split into two administrations per day). CX-4945 vehicle, phosphate buffer 25 mM pH 7.2 was administered to control mice (*n* = 2 per each experimental time). Administrations were performed with an oral gavage. Mice were euthanized at 2 h, 6 h and 24 h after the last administration for both treated and control mice to assess CK2 activity compared to control mice. Tumour samples were stored in liquid nitrogen until further processing.

#### 4.7.2. Longitudinal Studies with iCK2 (alone or in combination)

CX-4945 therapy was administered to tumour-bearing mice, every day or in alternated days, 150 mg/Kg/day [23] (Figures S2A,B). CX-4945 total dose for every day or alternated days treatment administered was 150 mg/Kg/day, split into two times per day (75 mg/kg at 8 h, and 75 mg/kg at 16 h) and dissolved in vehicle administration, which was phosphate buffer 25 mM pH 7.2. For the combined CX-4945 + TMZ therapy (Figure S2C), CX-4945 was administered every day whereas TMZ (60 mg/Kg) was dissolved in 10% DMSO in saline solution (0.9% NaCl) prepared as described in [5]. CX-4945 therapy was given until the end point of animal survival, when animals were euthanized because of welfare parameters. For this, animals were euthanized by cervical dislocation, the brain was removed and tumour resected. Samples were stored in liquid nitrogen until further processing for CK2 activity analysis. 

Regarding metronomic (every 6 days) administration protocol, eight doses of CX-4945 (150 mg/Kg; 75 mg/kg at 8 h and 75 mg/kg at 16 h) were administered to CX-4945 treated group (Figure S2D), eight doses of TMZ (60mg/Kg at 12 h) were administered to TMZ treated group (Figure S2E) and eight doses of CX-4945 (150 mg/Kg75 mg/kg at 8 h, and 75 mg/kg at 16 h) plus TMZ (60 mg/Kg at 12 h) were administered to CX and TMZ combined therapy group (Figure S2F). Control mice received TMZ and CX-4945 vehicles. In all cases, the maximum cumulative dose administered of CX-4945 was 1200 mg/Kg and of TMZ, 480 mg/kg. 

All treatments were administered using an oral gavage and the administration volume for CX-4945 and TMZ was the same that has been used in our group for the TMZ administration (10 µL/g weight animal) [5]. 

### 4.8. CK2 Activity Assay

CK2 activity was measured in 1–2 μg of lysate proteins (total protein extract of cell lysates) and 5–10 μg (total protein extract of brain mice samples), previously incubated 10 min at 30 °C with 0.1 mM CK2-tide (specific CK2 substrate peptide), by means of radioactive assays with gamma-33P ATP, in the presence of phosphorylation reaction mixture as described in [34]. 

### 4.9. MRI Acquisition

Magnetic resonance studies were carried out at the joint NMR facility of UAB and CIBER-BBN, Unit 25 of NANBIOSIS, with a 7T horizontal magnet (BioSpec 70/30, Bruker BioSpin, Ettlingen, Germany). GL261 tumour-bearing mice were screened by acquiring high resolution coronal T_2W_ images (TR/TE_eff_ = 4200/36 ms) using Rapid Acquisition with Relaxation Enhancement (RARE) sequence to detect brain tumour presence and monitor its evolution stage. The acquisition parameters were as follows: turbo factor, 8; field of view (FOV), 19.2 × 19.2 mm; matrix, 256 × 256 (75 × 75 μm/pixel); number of slices, 10; slice thickness (ST), 0.5 mm; inter-ST, 0.6 mm; number of averages (NA), 4; total acquisition time (TAT), 6 min and 43 s. 

### 4.10. Tissue Homogenization and Protein Extraction/Western Blot Analysis

Detailed information of the experimental procedures can be found in the Supplementary Materials file.

### 4.11. Statistical Analysis 

Variance homogeneity was assessed with the Levene’s test. A two-tailed Student’s *t*-test for independent measurements was used for comparisons, for samples of equal or different variances (depending on the Levene’s test result). Dixon’s and Grubb’s tests were used to detect outliers. The global evolution of tumour growth curves or body weight control was evaluated with the UNIANOVA test. Comparisons of survival rates were performed with the Log-Rank test. The significance level for all tests was *p <* 0.05.

## 5. Conclusions

CX-4945 has a noticeable effect in decreasing GL261 GBM cell viability and CK2 activity in vitro. Additionally, CK2 activity analysis confirmed that CX-4945 reached the target tissue in vivo. Notable differences in mice outcome were obtained with CX-4945 every day/alternated days (alone or combined with 3 cycles of TMZ), in comparison with metronomic administration of CX-4945 and/or TMZ, the highest survival rates being obtained with the metronomic combining TMZ + CX-4945 every 6 days.

An appealing explanation for this fact would be related with the immune system role in tumour response and the possible impairment of cytotoxic T-cell maturation cycle due to continued administration of CX-4945, which was overcome by the 6-day schedule metronomic administration. Accordingly, due care should be exercised when treating immunocompetent mice harbouring preclinical tumours with CX-4945 to ensure optimal results.

## Supplementary Materials

The following are available online at www.mdpi.com/1424-8247/10/1/24. Supplementary Materials and Methods S1.1: Cell Viability Assay, Supplementary Materials and Methods S1.2: Therapeutic Agent Preparations (CK2 Inhibitors and TMZ), Supplementary Materials and Methods S1.3: Tissue Homogenization and Protein Extraction, Supplementary Materials and Methods S1.4: Western Blot Analysis. Table S1: Tumor volume and body weight for mice before starting CX-4945 therapy every day, Table S2: Tumor volume and body weight for mice before starting CX-4945 therapy in alternated days, Table S3: Tumor volume and body weight for mice before starting combined TMZ+CX-4945 therapy, Table S4: Tumor volume and body weight for mice before starting metronomic therapy: CX-4945, TMZ, CX-4945 and TMZ, Table S5: Doses for CX-4945 and TMZ administration in MTD calculation experiments, Table S6: Symptoms and signals guidance to decide the MTD, Figure S1: Mice body weight (maximum tolerated dose (MTD) studies), Figure S2: Therapy administration scheme protocols, Figure S3: Weight averages of treated and control mice, Figure S4: MRI images of CX-4945 treated mice, Figure S5: Survival Kaplan-Meier curve for 3 cycles of TMZ vs. control.

## Abbreviation

APG	Apigenin
CK2	Protein Kinase CK2
CSCs	Cancer stem cells
CX-4945	5-(3-Chlorophenylamino)benzo[c][2,6] naphthyridine-8-carboxylic acid
CPA	Cyclophosphamide
DC_50_	Half maximal cell death concentration
DMSO	Dimethyl sulfoxide
EC_50_	Half maximal effective concentration,
GABRMN	Grup d’Aplicacions Biomèdiques de la RMN
GBM	Glioblastoma
IC_50_	Half maximal inhibitory concentration
iCK2	Protein Kinase CK2 inhibitors
JAK	Janus kinase
MGMT	O(6)-methylguanine-DNA methyltransferase
MTD	Maximum tolerated dose
p.i.	Post-inoculation
PIM-1	Proviral Integration of Moloney virus 1
STAT 3	Signal transducer and activator of transcription 3
TBB	4,5,6,7-Tetrabromobenzotriazole
TDB	Tetrabromodeoxyribofuranosyl-benzimidazole
TMZ	Temozolomide
WB	Western blot.
