# Supplementary material for: Targeting Protein Kinase CK2: Evaluating CX-4945 Potential for GL261 Glioblastoma Therapy in Immunocompetent Mice
Source: Pharmaceuticals (Basel). 2017 Feb 12;10(1):24. doi: 10.3390/ph10010024 (PMC5374428; doi:10.3390/ph10010024)
Supplement: Supplementary file 1 [file pharmaceuticals-10-00024-s001.pdf]

Supplementary Materials: Targeting Protein Kinase Ck2: Evaluating Cx-4945 Potential for Gl261 Glioblastoma Therapy in Immunocompetent Mice

Laura Ferrer-Font, Lucia Villamañan, Nuria Arias-Ramos, Jordi Vilardell, Maria Plana, Maria Ruzzene, Lorenzo A. Pinna, Emilio Itarte, Carles Arús and Ana Paula Candiota

S1. Supplementary Materials and Methods

S1.1. Cell Viability Assay

GL261 cells were plated at 5000 cells per well in 96-well multiwell plates (Sigma Aldrich, Madrid, Spain). Cells were allowed to adhere for 24 h before drugs were added to the medium at increasing concentrations: for Temozolomide (TMZ), 0 μM, 0.8 μM, 4 μM, 20 μM, 100 μM, 200 μM, 500 μM, 1000 μM, 5000 μM and 10,000 μM, apigenin (APG) and 4,5,6,7-Tetrabromobenzotriazole (TBB): 0 μM, 0.8 μM, 4 μM, 20 μM, 100 μM, 200 μM and 500 μM, and 5-(3-Chlorophenylamino)benzo[c][2,6] naphthyridine-8-carboxylic acid (CX-4945): 0 μM, 0.2 μM, 2 μM, 5 μM, 20 μM, 50 μM, 100 μM, 200 μM and 500 μM. Controls in each plate included cell culture RPMI (Roswell Park Memorial Institute) medium and dimethyl sulfoxide (DMSO) (0.4% for CX-4945, TBB and APG, and 0.8% for TMZ).
Drug-treated and control wells were run in triplicate. After 72 h of drug exposure, cell viability was measured using 2,3-bis-(2-methoxy-4-nitro-5-sulfophenyl)-2H-tetrazolium-5-carboxanilide (XTT) Assay (Sigma Aldrich, Madrid, Spain) as per the manufacturer's instructions. DMSO-treated wells were considered as "100% viability" for each treatment plate. In the case of combined TMZ and CX-4945 treatment, 3-(4,5-dimethylthiazol-2-yl)-2,5-diphenyltetrazolium bromide (MTT) assay (Sigma Aldrich) was used for cell viability assay. Two compound combinations were used: CX-4945 30 μM plus TMZ 1 mM, and CX-4945 50 μM plus TMZ 1.5 mM, which were compared to control (medium and DMSO) and TMZ or CX-4945 alone. Controls included RPMI medium and DMSO (0.8%).

S1.2. Therapeutic Agent Preparations (CK2 Inhibitors and TMZ)

CX-4945 sodium salt (Glixx Laboratories, Southborough, MA, USA) was dissolved in 0.4% DMSO (cell experiments), or in phosphate buffer 25 mM pH 7.2 (in vivo studies), as described in [1]. APG (Sigma-Aldrich) and TBB (Calbiochem, Merck KGaA, Darmstadt, Germany), used for cell experiments, were dissolved in 0.4% DMSO. TMZ (Sigma-Aldrich) was dissolved in 0.8% DMSO for cell experiments, and in 10% DMSO in saline solution (0.9% NaCl) for in vivo experiments.

S1.3. Tissue Homogenization and Protein Extraction

Tissue samples were weighted and 250 μL of cold lysis buffer for each 100 mg of tissue was added (cold lysis buffer: 20 mM Tris-HCl pH 7.4, 150 mM NaCl, 1 mM EDTA, 0.2% sodium deoxycholate, 2× proteases inhibitor EDTA free (Roche, Madrid, Spain)), 1× phosphatases inhibitors (Sigma-Aldrich): Phosphatase inhibitor cocktail 2 (Reference P5726), Phosphatase inhibitor cocktail 3 (Reference P0044) and 1% triton-x-100 (Sigma-Aldrich). Samples were homogenized with a 20 G needle 10 times and with a 26 G needle 10 more times. Sonication (Fisher Sonic Dismembrator Model 300, Thermo Fisher Scientific, Waltham, MA, USA) was performed five times for 5-s intervals at 30% amplification. After remaining 30 min on ice, the lysate was centrifuged at 25,000× *g* for 20 min at 4 °C. Supernatants were used for WB and CK2 activity analysis.

S1.4. Western Blot Analysis

GL261 cells were lysed as described in [2]. Protein concentration was determined by Bradford method [3] and equal amounts of protein (25 μg) were loaded on 11% Sodium Dodecyl Sulfate Polyacrylamide Gel Electrophoresis (SDS-PAGE), blotted on polyvinylidene fluoride (PVDF) Immobilon-P membranes (Millipore, Darmstadt, Germany), and immunodetected with the

corresponding antibodies using a chemiluminescent detection method. Chemiluminescent signal obtained was quantified in a Kodak Image Station 440MMPRO (Eastman Kodak Company, Rochester, NY, USA) and analyzed with the Kodak 1DImage software. For tumor samples, 40 µg of tissue homogenate protein was loaded.

Table S1. Average ± standard deviation (AV ± SD) for tumor volume (mm^3) and body weight (g) for mice before starting CX-4945 therapy every day (day 11 post-inoculation). No significant differences ($p > 0.05$) were found between CX-4945 every day treated group ($n = 6$) and control mice ($n = 6$) neither for tumor volumes, nor for mice body weight. Student's t-test applied.

DAY 11 (Tumor Volume and Body Weight)								
	**Mice**	**C940**	**C942**	**C943**	**C944**	**C945**	**C946**	**AV ± SD**
**CONTROL**	**Weight**	21.7	21.2	20.4	22.6	19.5	20.9	21.1 ± 1.1
	**Volume**	11.7	12.4	17.9	22.9	12.5	10.9	14.7 ± 4.7
	**Mice**	**C947**	**C948**	**C949**	**C951**	**C952**	**C953**	**AV ± SD**
**CX-4945 every day**	**Weight**	21.0	21.8	22.1	22.3	22.6	22.3	22.0 ± 0.6
	**Volume**	17.7	17.9	17.3	25.4	11.5	13.3	17.2 ± 4.8

Table S2. Average ± standard deviation (AV ± SD) for tumor volume (mm^3) and body weight (g) for mice before starting CX-4945 therapy in alternated days (day 11 post-inoculation). No significant differences ($p > 0.05$) were found between CX-4945 alternated days treated group ($n = 6$) and control mice ($n = 6$) neither for tumor volumes, nor for mice body weight. Student's *t*-test applied.

DAY 11 (Tumor Volume and Weight)								
	**Mice**	**C955**	**C956**	**C957**	**C958**	**C960**	**C965**	**AV ± SD**
**CONTROL**	**Weight**	25.5	20.0	19.5	22.0	20.1	22.7	21.6 ± 2.3
	**Volume**	10.7	7.1	22.6	4.9	15.8	11.4	12.1 ± 6.4
	**Mice**	**C961**	**C962**	**C963**	**C964**	**C067**	**C950**	**AV ± SD**
**CX-4945 alternated days**	**Weight**	21.0	19.5	22.3	20.8	20.8	21.7	21.0 ± 0.9
	**Volume**	22.2	14.8	11.1	18.2	10.9	11.9	14.9 ± 4.6

Table S3. Average ± standard deviation (AV ± SD) for tumor volume (mm^3) and body weight (g) for mice before starting combined Temozolomide (TMZ)+CX-4945 therapy (day 11 post-inoculation). No significant differences ($p > 0.05$) were found between 3 cycles TMZ + CX-4945 every day treated group ($n = 6$) and control mice ($n = 6$) neither for tumor volumes, nor for mice body weight. Student's *t*-test applied.

DAY 11 (Tumor Volume and Weight)								
	**Mice**	**C991**	**C992**	**C996**	**C997**	**C998**	**C999**	**AV ± SD**
**CONTROL**	**Weight**	21.3	21.9	21.1	21.4	20.0	19.6	20.8 ± 0.9
	**Volume**	10.6	23.9	8.8	6.8	24.0	8.0	13.7 ± 8.0
	**Mice**	**C984**	**C985**	**C988**	**C990**	**C994**	**C995**	**AV ± SD**
**TMZ and CX-4945**	**Weight**	22.4	22.1	21.7	22.6	21.5	24.4	22.5 ± 1.0
	**Volume**	13.0	14.6	9.5	8.8	22.5	11.6	13.3 ± 5.0

Table S4. Average ± standard deviation (AV ± SD) for tumor volume (mm^3) and body weight (g) for mice before starting metronomic therapy: CX-4945, TMZ, CX-4945 and TMZ, and control mice (day 10 post-inoculation). No significant differences ($p > 0.05$) were found between the different groups ($n = 6$) neither for tumor volumes, nor for mice body weight. Student's *t*-test applied.

DAY 10 (Tumor Volume and Weight)								
**CX**	**Mice**	**C1144**	**C1158**	**C1147**	**C1148**	**C1149**	**C1150**	**AV ± SD**
	**Weight**	20.6	20.6	20.6	20.4	19.9	20.8	20.5 ± 0.3
	**Volume**	5.3	5.1	6.5	7.5	2.3	3.0	5.0 ± 2.0
**CX+TMZ**	**Mice**	**C1151**	**C1152**	**C1153**	**C1154**	**C1155**	**C1156**	**AV ± SD**
	**Weight**	21.9	20.7	21.0	20.1	22.5	24.1	21.7 ± 1.4
	**Volume**	5.9	7.7	3.1	5.2	4.6	2.9	4.9 ± 1.8
**TMZ**	**Mice**	**C1166**	**C1167**	**C1168**	**C1169**	**C1170**	**C1171**	**AV ± SD**
	**Weight**	19.7	20.7	22.0	21.1	17.1	21.0	20.3 ± 1.7
	**Volume**	4.9	5.3	5.8	6.9	5.3	8.0	6.0 ± 1.2
**CONTROL**	**Mice**	**C1157**	**C1145**	**C1160**	**C1161**	**C1162**	**C1165**	**AV ± SD**
	**Weight**	20.9	20.2	21.1	21.1	21.1	20.0	20.7 ± 0.5
	**Volume**	5.2	4.1	6.9	3.6	10.1	7.1	6.2 ± 2.4

Table S5. Doses for CX-4945 and TMZ administration in maximum tolerated dose (MTD) calculation experiments. The final volume administration and doses were adjusted to actual animal weights.

**Day**	**0**	**4**	**8**	**12**	**16**	**20**
**CX-4945 (mg/Kg)**	150	300	600	1200	2400	4800
**TMZ (mg/Kg)**	60	120	240	480	920	1890

Table S6. Symptoms and signals guidance to decide the MTD. Adapted from [4]. If at least two parameters for endpoint are detected, there is indication of adverse side effects and further dose increasing is discouraged.

**Parameter for Endpoint**	**Means of Verification**
Weight loss of above 20% regarding the previous register	Scale readings
Marked piloerection	Piloerection detected during animal observation
Animal shows subdued behaviour patterns even when provoked	Apathic behaviour during weighting procedure, in comparison with control animals.
Intermittent or persistent tremors	Observation of animals before and after weighting procedure

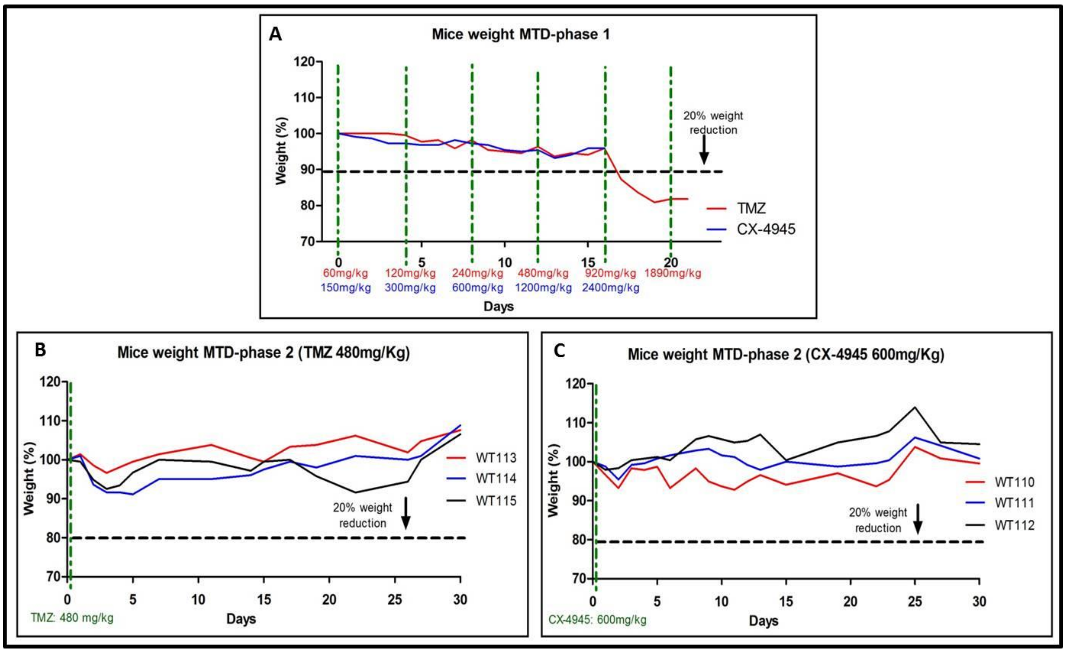

Figure 1. Mice body weight (maximum tolerated dose (MTD) studies). (**A**) Body weight of mice treated with increasing doses of Temozolomide (TMZ) (red line) and CX-4945 (blue line); (**B**) Body weight of mice treated with TMZ single dose (480 mg/kg) ($n = 3$) and (**C**) Body weight of mice treated two times a day with CX-4945 (600 mg/kg total dose) ($n = 3$). In all cases, the weight is expressed in %, considering 100% as the initial weight, and the dashed black line indicates the 20% weight reduction point. See main article text for further details.

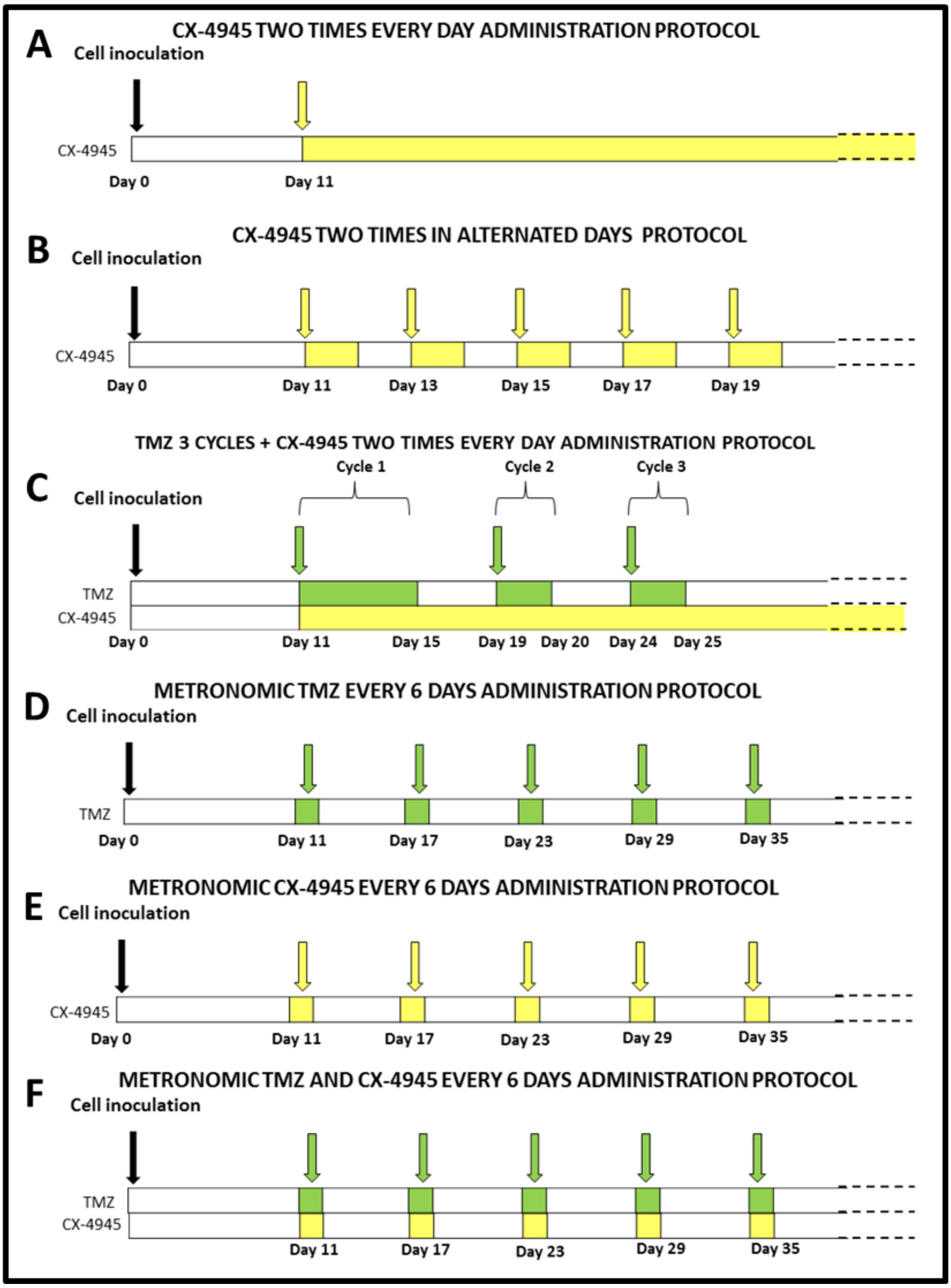

Figure 2. Therapy administration scheme protocols (**A**) for every day CX-4945 (150 mg/kg) administration (75 mg/kg at 8 h and 75 mg/kg at 16 h) (**B**) for alternated days CX-4945 (150 mg/kg) administration protocol (75 mg/kg at 8 h and 75 mg/kg at 16 h) (**C**) for TMZ + CX-4945 administration protocol. TMZ 60 mg/kg was administered at days 11–15, 19–20 and 24–25 post-inoculation and 150 mg/kg of CX-4945 (75 mg/kg at 8 h and 75 mg/kg at 16 h) (**D**) for metronomic TMZ (60 mg/Kg) every 6 days protocol (**E**) for metronomic CX-4945 (75 mg/kg at 8 h and 75 mg/kg at 16 h) every 6 days protocol and (**F**) for metronomic TMZ + CX-4945 every six days administration protocol. TMZ 60 mg/kg and 150 mg/kg of CX-4945 were administered: CX-4945 75 mg/kg at 8 h, TMZ 60 mg/Kg at 12 h and CX-4945 75 mg/kg at 16 h). In all cases, treatment started at day 11 post-inoculation.

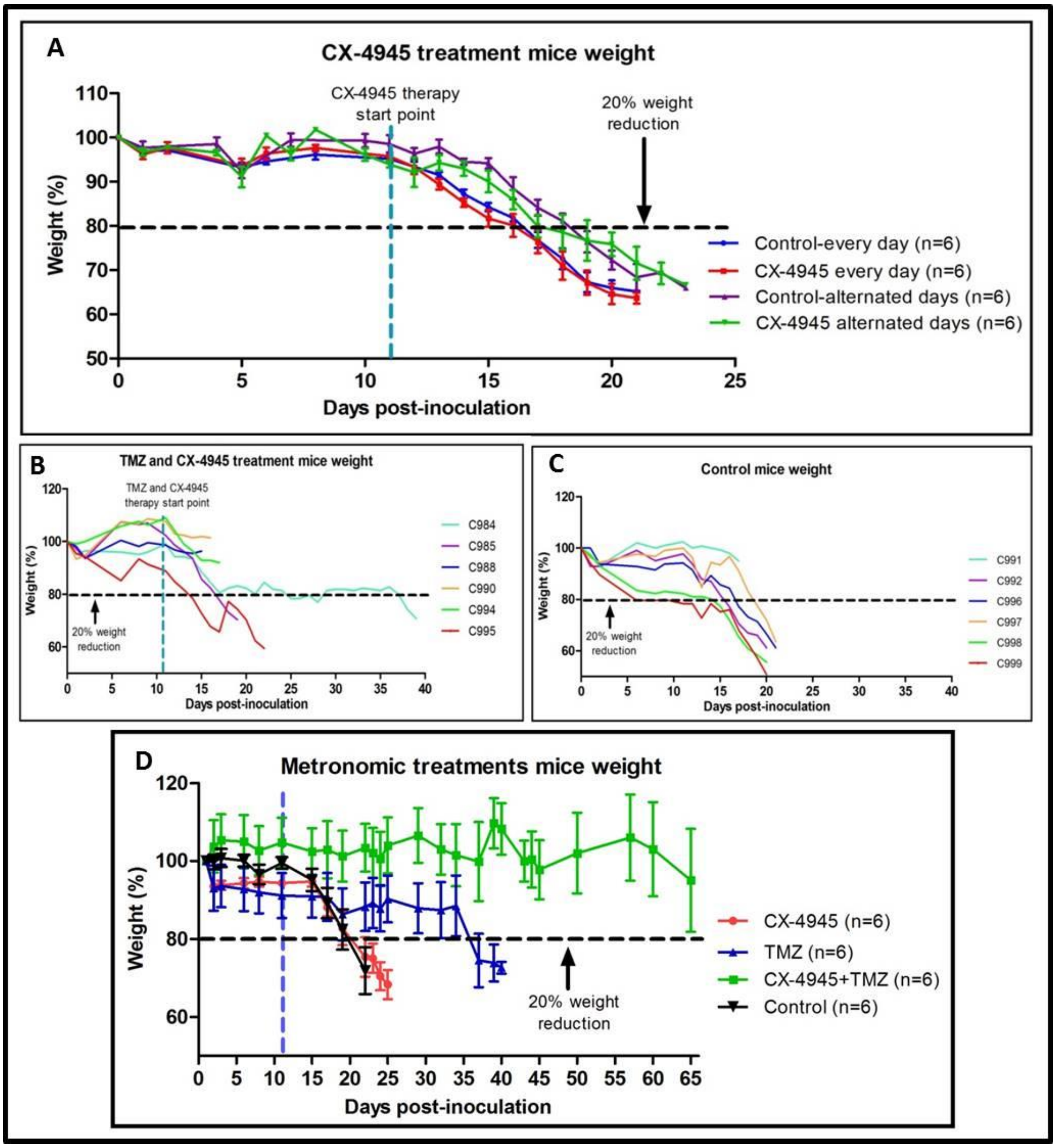

Figure 3. Weight averages of treated and control mice. (**A**) Weight (average ± SD) of mice treated two times every day with CX-4945 (n = 6, red line) and control vehicle (n = 6, blue line), and for mice treated two times a day in alternated days with CX-4945 (n = 6, green line) and control vehicle (n = 6, purple line). The dashed blue line indicates CX-4945 therapy start point. No differences were observed between groups ($p > 0.05$); (**B**) Weights of individual mice treated with a combination of TMZ cycles (5-2-2) [5] and CX-4945 two times every day (n = 6) until death or euthanasia for ethical reasons. The dashed blue line indicates TMZ and CX-4945 therapy start point. Case C984 was considered an outlier according to Grubbs' and Dixon's tests ($p < 0.05$); (**C**) Body weight of each control mice (controls of mice represented in B) until death or euthanasia for ethical reasons. Administration of vehicles: phosphate buffer two times a day (CX-4945 vehicle) and 10% DMSO solution in 0.9% NaCl (TMZ vehicle) in 3 cycles, were performed. In all cases, the weight is expressed in %, assuming that at day 0 the initial weight corresponds to 100%. The dashed black line indicates the 20% weight reduction point; (**D**) Weight (average ± SD) of mice treated with CX-4945 metronomic treatment (n = 6, red line), of mice treated with TMZ metronomic treatment (n = 6, blue line), of mice treated with CX-4945 and TMZ metronomic treatment (n = 6, green line) and control mice (n = 6, blue line). The dashed blue line indicates therapies start point. Significant differences were observed between all groups (control vs. treated groups and different treatments between them ($p < 0.05$)).

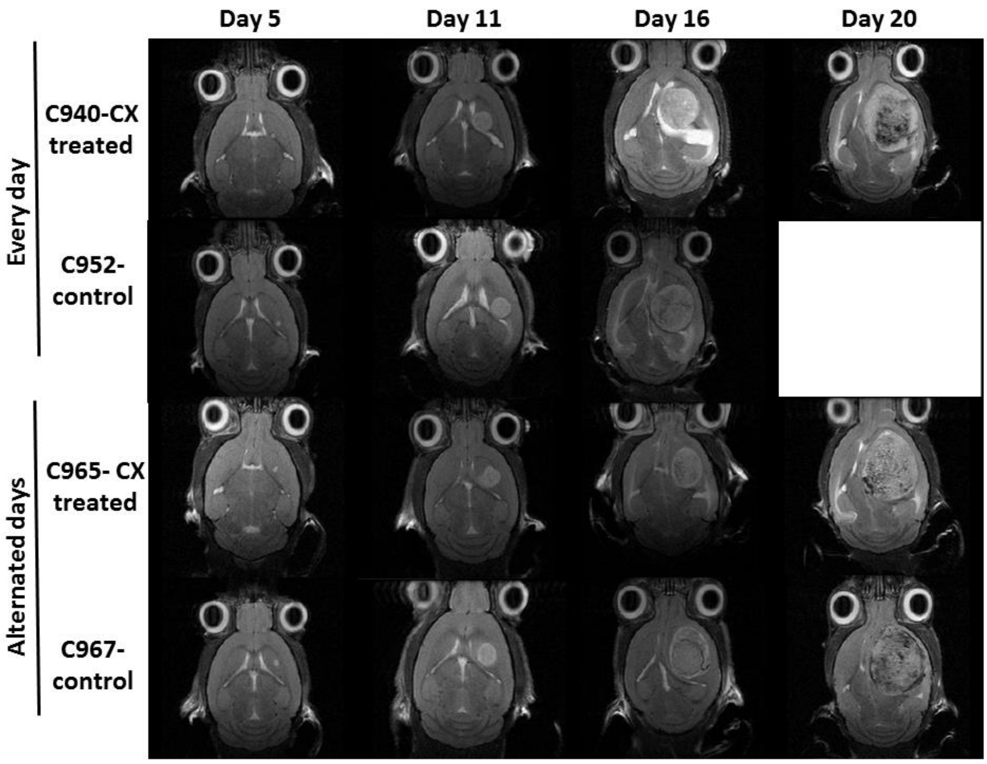

Figure 4. Magnetic resonance imaging (MRI) images of CX-4945 treated mice. Follow up of tumor volume evolution by T2w MRI axial images of CX-4945- treated tumor bearing mice (at days 5, 11, 16 and 20 post-inoculation). C940: treated with CX-4945 every day, C952: control of every day treatment (phosphate buffer CX-4945 vehicle administration), C965: CX-4945 treated in alternated days and C967: control for alternated days (vehicle administration). CX-4945 dosage was 150mg/kg split into two times per day (75 mg/kg 8 h and 75 mg/kg 16 h). MRI is not displayed for C952 day 20 because this mouse was found dead the day 17 post-inoculation. C940 was euthanized the day 20 post-inoculation for ethical reasons, and C965 and C967 were euthanized the days 21 and 20, respectively. Cxxxx corresponds to a unique alpha-numeric animal identifier code in the GABRMN group.

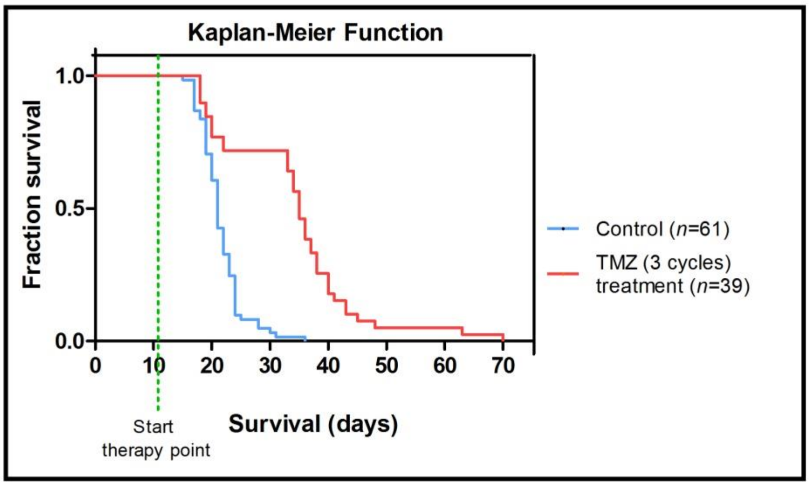

Figure 5. Survival Kaplan-Meier curve for 3 cycles of TMZ vs. control. Control mice ($n = 61$, blue line) and TMZ treatment ($n = 39$, red line). Survival rate average was 21.5 ± 3.7 days for control mice and 33.9 ± 11.7 days for TMZ (3 cycles) treated mice. Significant differences were found between groups ($p < 0.05$) when

comparing control mice with TMZ treated mice. The dashed green line indicates the therapy start point. Results for control and TMZ treatment extracted from [5] and unpublished data.

References

1. Siddiqui-Jain, A.; Drygin, D.; Streiner, N.; Chua, P.; Pierre, F.; O'Brien, S.E.; Bliesath, J.; Omori, M.; Huser, N.; Ho, C.; et al. CX-4945, an orally bioavailable selective inhibitor of protein kinase CK2, inhibits prosurvival and angiogenic signaling and exhibits antitumour efficacy. *Cancer Res*. **2010**, *70*, 10288–10298.
2. Di Maira, G.; Salvi, M.; Arrigoni, G.; Marin, O.; Sarno, S.; Brustolon, F.; Pinna, L.A.; Ruzzene, M. Protein kinase CK2 phosphorylates and upregulates Akt/PKB. *Cell Death Differ*. **2005**, *12*, 668–677.
3. Bradford, M.M. A rapid and sensitive method for the quantitation of microgram quantities of protein utilizing the principle of protein-dye binding. *Anal. Biochem.* **1976**, *72*, 248–254.
4. LASA and NC3Rs, Guidance on dose level selection for regulatory general toxicology studies for pharmaceuticals, R.a.R.o.A.i.R. Laboratory Animal Science Association & National Centre for the Replacement, Editor. 2009: London. Available online: http://www.lasa.co.uk/pdf/lasa-nc3rsdoselevelselection.pdf (accesed on 12 February 2017).
5. Delgado-Goni, T.; Julia-Sape, M.; Candiota, A.P.; Pumarola, M.; Arus, C. Molecular imaging coupled to pattern recognition distinguishes response to temozolomide in preclinical glioblastoma. *NMR Biomed*. **2014,** *27*, 1333–1345.
